# Development and application of a monoclonal antibody-based blocking ELISA for detection of antibodies to Tembusu virus in multiple poultry species

**DOI:** 10.1186/s12917-018-1537-6

**Published:** 2018-06-25

**Authors:** Lijiao Zhang, Zhanhong Li, Huan Jin, Xueying Hu, Jingliang Su

**Affiliations:** 1 Key Laboratory of Animal Epidemiology of the Ministry of Agriculture, College of Veterinary Medicine, China Agricultural University, Beijing, 100193 China; 2 College of Veterinary Medicine, Huazhong Agricultural University, Wuhan, 430070 China

**Keywords:** Duck, Tembusu virus, Flavivirus, Monoclonal antibody, Blocking ELISA

## Abstract

**Background:**

Tembusu virus (TMUV) is a member of the genus *Flavivirus*. Outbreak of this virus infection in duck flocks was first observed in China in April 2010, causing severe egg drop and neurological signs in laying ducks. Recently reported duck infections in southeastern Asia highlighted the need for well-validated diagnostic methods of TMUV surveillance to understand its epidemiological characteristics and maintenance in nature. Several enzyme-linked immunosorbent assays (ELISAs) for the detection of TMUV infection have been reported, but none have been applied to high-throughput diagnostics.

**Results:**

In this study, a monoclonal antibody (MAb) against TMUV was generated and characterized. MAb 9E4 was shown to bind specifically to a disulfide bond-dependent epitope on the domain I/II of TMUV E protein, and a blocking ELISA was established based on this MAb. The cut-off percentage inhibition value for negative sera was set at 30%. By comparison with the virus neutralization test, the specificity and sensitivity of the blocking ELISA were 96.37% and 100%, respectively, and the kappa value was 0.966, based on 416 serum samples collected from both experimentally and clinically infected ducks, geese and chickens. A good correlation (r^2^ = 07998, *P* < 0.001) was observed between the blocking ELISA and plaque reduction neutralization test (PRNT) titers. Using archived duck serum samples collected between 2009 and 2015, the seroprevalence in duck flocks raised in Northern China was estimated by blocking ELISA.

**Conclusions:**

Our MAb-based blocking ELISA provides a reliable and rapid diagnostic tool for serological monitoring of TMUV infection and evaluation of immune status following TMUV vaccination in multiple poultry species.

**Electronic supplementary material:**

The online version of this article (10.1186/s12917-018-1537-6) contains supplementary material, which is available to authorized users.

## Background

Tembusu virus (TMUV) is a mosquito-borne virus that belongs to the genus *Flavivirus* within the family *Flaviviridae*. The virus was originally isolated from mosquitoes in Malaysia in 1953 and the first animal infection case was reported in a 4-week-old broiler chicken flock in Perak State, characterized by encephalitis, growth retardation and increased blood glucose levels [1]. In April 2010, a severe outbreak of duck TMUV infection causing egg drop and signs of central nerve system involvement was reported in China [2]. Thereafter, the disease spread diffusely throughout duck-producing regions, involving ducks, geese and even laying chickens [3–5]. The recent emergence of TMUV infections in duck flocks in Malaysia and Thailand provided warnings of the increasing impact on animal health [6, 7]. In addition to domestic birds, TMUV has been occasionally isolated from mosquitos, pigeons and house sparrows in the vicinity of duck farms in China [8–10], suggesting that wild birds may serve as a natural reservoir, carrying and disseminating the virus. However, the epidemiological characteristics and maintenance in nature of TMUV remain unclear, largely owing to the absence of convenient, sensitive and specific diagnostic tests.

Currently, identification of TMUV infection in ducks is based on isolation of the virus or detection of the viral nucleic acid by reverse transcriptase PCR. The diagnostic efficiency is largely dependent on the longevity of the virus in samples, which is closely related to the time when the samples are collected after infection. Since serologic evidence of infection may present rapidly and last long after infection, detection of TMUV-specific antibodies in serum samples could provide a convenient way to determine virus infection in animal populations. In this respect, enzyme-linked immunosorbent assays (ELISAs) are much more suitable for vaccination assessment and epidemiological analyses involving a large number of samples. Several ELISAs for the detection of TMUV infection have been reported recently [11–13], but none of them have been applied to large-scale clinical serum sample testing. We have recently described the uses of a *Flavivirus* group-specific monoclonal antibody-based blocking ELISA for detection of antibody responses in ducks immunized with an inactivated TMUV vaccine under laboratory conditions [14]. The drawback of this ELISA is the cross-reactivity with antibodies to other flaviviruses that infect animals in the field. In this paper, we describe the development of a blocking ELISA based on a TMUV-specific MAb and evaluate its potential application for high throughput of clinical serum samples.

## Methods

### Preparation of virus antigen

Duck TMUV strain JXSP was isolated from an infected duck flock as described previously [9]. After the initial two passages in duck embryos, the virus was propagated in baby hamster kidney cells (BHK-21) cells and used as stock virus (designated as JXSP_2–4_) for antigen preparation or virus neutralization test (VNT). For antigen preparation, BHK-21 cells were grown and infected with JXSP_2–4_ at a multiplicity of infection (MOI) of 0.001. When the cytopathic effect (CPE) reached approximately 75%, the infected supernatant was harvested by three freeze-thaw cycles, followed by centrifugation at 10,000×g for 45 min at 4 °C. To inactivate the virus, beta-propiolactone (BPL) (FERAK Berlin Gmbh, Berlin, Germany; NMR ≥ 98.5%) was added to the clarified virus suspensions to the final concentration of 1: 4000 and incubated at 4 °C for 24 h [14]. Virus particles were pelleted by ultracentrifugation at 160,000×g for 2.5 h at 4 °C, then resuspended in PBS and stored at − 80 °C until use.

### Production of monoclonal antibody

Five female six-week-old BALB/c mice (Vitalriver, China) were injected subcutaneously with 100 μg of BPL-inactivated virus antigen emulsified with complete Freund’s adjuvant (Sigma-Aldrich, St Louis, MO), followed by two subcutaneous boosters of the same antigen with incomplete Freund’s adjuvant and one intraperitoneal inoculation of the antigen without adjuvant at ten days intervals. After the fourth inoculation, mouse spleen cells were harvested to prepare hybridomas using the standard method. Hybridomas secreting antibody against TMUV were screened by indirect ELISA, and sub-cloned three times by limiting dilution. The supernatant of the hybridoma culture was collected for immunoglobulin isotyping using the Mouse Monoclonal Antibody Isotyping Kit (Sigma-Alrich) according to the manufacturer’s instructions. The selected hybridoma was inoculated into BALB/c mice and ascitic fluid was purified by saturated ammonium sulfate (SAS) precipitation as described [15].

### Western blot analysis

To investigate the antigen binding of the generated MAbs, virus concentrated by ultracentrifugation was resuspended in reducing or non-reducing lane marker sample buffer (Thermo scientific, USA) and boiled for 6 min before SDS-PAGE separation. The separated proteins were transferred onto a PVDF (Polyvinylidene Fluoride) membrane, followed by incubation in blocking buffer (5% skim milk in PBS with 0.05% Tween-20) overnight at 4 °C. After washing, the protein was probed with the MAb and horseradish peroxidase (HRP)-conjugated goat anti-mouse IgG at a dilution of 1:5000. The signal was developed with chemiluminescence substrate (ECL reagent, Cwbiotech, Beijing, China). To further analyze the MAb binding domain, full length E protein of TMUV, domain I/II and domain III of E protein were individually expressed in *E. coli* using the pET32α vector (see Additional file 1). Purified and renatured recombinant protein was separated by SDS-PAGE under non-reducing condition and analyzed using the generated MAbs by Western blot as described above.

### Immunofluorescence assay and immunochemistry

For the immunofluorescence assay (IFA), BHK-21 cells were cultured in 96-well-plates. Cells were infected with JXSP_2–4_, Japanese encephalitis virus or duck-origin Batai virus at an MOI of 0.001 for 1 h and maintained in DMEM with 2% FBS for 36 h in a CO_2_ incubator. The cells were then fixed with an ice-cold acetone/methanol (1:1) mixture for 20 min at room temperature. After washing three times with PBS, 200 μL of the blocking buffer was added and incubated at 37 °C for 30 min. Wells were then gently washed with PBS, the hybridoma culture supernatant or diluted murine ascitic fluid was added and incubated at 37 °C for 45 min. Wells were washed and FITC-conjugated goat anti-mouse IgG (Eathox, USA) was added at a dilution of 1:800, followed by 30 min incubation at 37 °C. After three times washes, nuclei of the cells were stained with DAPI (Solarbio, China) for 10 min at room temperature. Wells were washed again and observed under fluorescence microscopy. For immunochemistry, BHK-21 cells were cultured on coverslips in a 24-well-plate, infected as described above and fixed with 4% paraformaldehyde for 30 min. Paraformaldehyde was removed by washing with PBS and cells were stained with the MAb as previously described [16].

### Virus neutralization test

The plaque reduction neutralization test (PRNT) was performed in 12-well plates as previously described with slight modification [17] to verify the presence of TMUV-specific antibodies in serum samples and to quantitate antibody titers. Briefly, sera were inactivated at 56 °C for 30 min and serially diluted with DMEM. The stock virus JXSP_2–4_ was diluted, mixed with an equal volume of diluted serum and incubated at 37 °C for 1 h. The mixture was transferred to BHK-21 cell monolayers in a 12-well plate in duplicate to a concentration of 100 plaque forming units (PFU) of infectious virus per well. After incubation at 37 °C for 1 h, the supernatant was removed and overlaid with 2.5 mL DMEM containing 1.0% (*w*/*v*) LMP agarose and 2% FBS. Following 3 days of incubation at 37 °C, infected cells were stained with 0.03% (w/v) neutral red and plaques were counted. Compared with the negative serum control, tested samples which showed more than 50% plaque reduction (PRNT_50_) at a five-fold dilution were considered positive. The PRNT_50_ titer for each sample tested was determined by identifying the well containing the highest dilution of serum with a plaque count < 50% of the average negative serum controls.

### Development of MAb-based blocking ELISA

Ninety-six-well microtiter plates (Costar, USA) were coated with 1 μg inactivated TMUV antigen (corresponding to 1.7 × 10^5^ PFU of the infectious virus) per well in 100 μL of 0.05 M carbonate-bicarbonate buffer (pH 9.6) and incubated at 4 °C overnight. The plate was washed three times with PBST (0.05% Tween in PBS, *v*/v) and 200 μL of blocking buffer was added for 2 h at 37 °C. The plate was washed three times and 100 μL of duck serum diluted with blocking buffer was added. After incubation at 37 °C for 1 h, the plate was washed with PBST, then 100 μL of SAS-purified ascitic MAb (0.85 μg/mL) was added and incubated at 37 °C for 1 h. After three washes, HRP conjugated goat anti-mouse IgG (1:5000 dilution) was added and incubated for 45 min at 37 °C. The plate was thoroughly washed and 100 μL of TMB substrate solution (3,3′,5,5′-tetramethyl benzidine 0.24 mg/mL and 0.003% H_2_O_2_) was added. Following incubation at room temperature in the dark for 15 min, the chromogenic reaction was stopped with 50 μL of 0.5 M sulfuric acid and optical density (OD) was measured at 450 nm in a microplate reader (Thermo Scientific, USA). Control wells without primary serum or without MAb were also prepared. The percentage inhibition (PI) of each test sample was calculated by the following formula: PI (%) = [(OD_0_-OD_sample_)/(OD_0_-OD_100_)] × 100% as described, where OD_0_ was the mean optical density of the negative control serum (0% inhibition), OD_100_ was the background optical density (100% inhibition) [17].

To determine the optimal dilution of serum samples to be tested, five TMUV antibody-negative sera and five positive sera were serially diluted and analyzed by blocking ELISA (bELISA). Subsequently, 400 duck serum samples collected from TMUV-free farms were tested for determination of the cut-off value. These sera were confirmed to be TMUV-specific antibody-negative by PRNT.

### Validation of the blocking ELISA

To validate the bELISA, the specificity and sensitivity were determined. Serum samples with antibodies against duck enteritis virus (DEV), duck hepatitis virus (DHV), Newcastle disease virus (NDV), duck reovirus (DRV), egg drop syndrome virus (EDSV) and avian influenza virus (AIV) subtypes H5 and H9 were analyzed with bELISA to evaluate cross-reactivity.

Two experiments were further performed to evaluate the bELISA. In the first experiment, one-day-old ducklings purchased from a TMUV-free farm were kept in positive pressure specific-pathogen-free (SPF) chicken isolators with ad libitum access to feed and water. Ten ducklings were infected subcutaneously with 3 × 10^5^ PFU of a moderately attenuated TMUV in 0.5 mL at the age of 7 days. Negative control ducklings were kept in a separate isolator. Serum samples were collected on days 2, 4, 7, 14 and 21 post-vaccination for detection of antibody titers by both PRNT and bELISA. Each sample was initially diluted five-fold and then subjected to doubling dilution. In the second experiment, 340 duck and 46 goose serum samples collected from flocks on farms with a history of TMUV infection in the previous two years were tested by bELISA and PRNT in parallel. Meanwhile, 10 chicken sera collected from SPF chickens immunized twice with inactivated TMUV were also tested. Compared with the PRNT, the sensitivity and specificity of the bELISA were calculated according to the following formulae: sensitivity = true positives × 100/ (true positives + false negatives), specificity = true negatives × 100/ (true negatives + false positives).

### Field samples

A total of 2349 serum samples belonging to 17 flocks in Northern China were tested by bELISA for antibodies to TMUV. These sera were submitted to our laboratory for assessment of AIV vaccination from 2009 to 2015 by the duck farm owners (Table 1).

**Table 1:** Tembusu virus (TMUV) seroprevalence in domestic ducks in Northern China, 2009–2015

Sampling date	Sampling site	Species	No. sera	b-ELISA positive	Positive rate
2009	Hebei	laying duck	120	0	0%
2009	Beijing	laying duck	80	0	0%
2011.03	Hebei	layingduck^a^	289	289	100%
2011.03	Beijing	breeding duck^a^	80	80	100%
2011.03	Shandong	breeding duck^a^	60	60	100%
2012. 11	Beijing	laying duck^b^	152	18	11.84%
2012. 12	Beijing	laying duck^b^	96	9	9.38%
2013. 01	Beijing	laying duck^b^	122	12	9.83%
2013.03	Beijing	table duckling^b^	90	0	0%
2013. 04	Beijing	table duckling^b^	176	0	0%
2013.06	Beijing	laying duck^b^	180	6	3.33%
2013.08	Beijing	laying duck^b^	190	25	13.16%
2013.12	Beijing	table duckling^b^	106	4	3.77%
2015.05	Beijing	table duckling^b^	50	0	0%
2015.09	Hebei	breeding duck^b^	80	0	0%
2015.12	Hebei	breeding duck^c^	241	79	32.78%
2015.12	Beijing	breeding duck^c^	237	226	95.36%

^a^The sera were from the animals suffered from DTMUV infection in December 2010

^b^The sera were from the animals with no history of DTMUV infection and immunization

^c^The sera were from the animals immunized with autogenous vaccine of DTMUV within one year

## Results

### Characterization of the monoclonal antibody

**Fig. 1:**
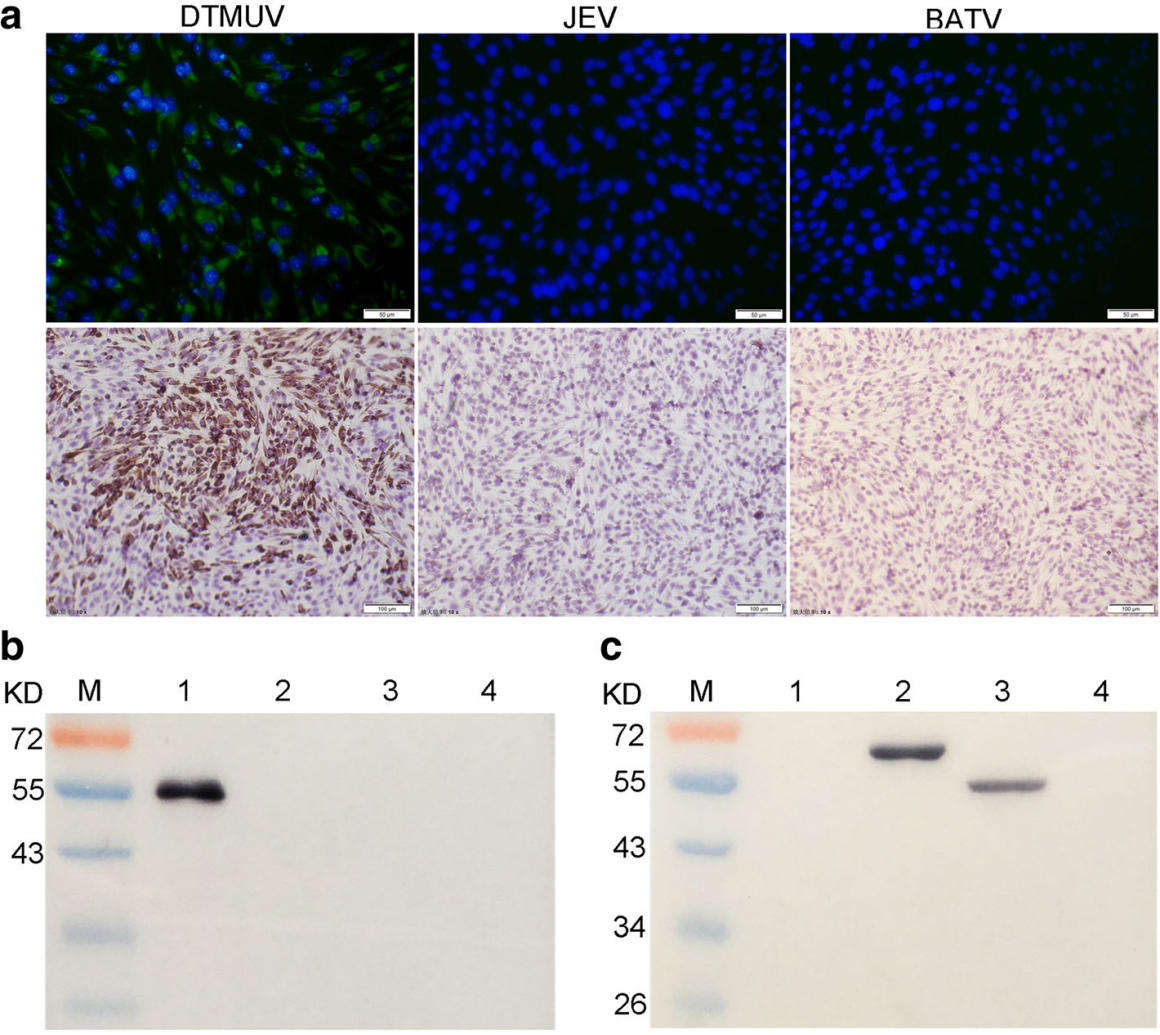
Characterization of the MAb 9E4 against TMUV. **a** Indirect immunofluorescence assay and immunocytochemistry of MAb 9E4 against duck TMUV, Japanese encephalitis virus and batai virus-infected BHK-21 cells. **b** Western blot analysis of MAb 9E4 reactivity against the virus particle. 1&2: Virus particle under non-reducing conditions or reducing conditions; 3&4: BHK-21 cell lysates under non-reducing conditions or reducing conditions; **c** Western blot analysis of MAb 9E4 reactivity against disulfide bonds reformed recombinant expression protein under non-reducing condition. 1: pET 32α tag protein; 2: E ectodomain; 3: E domain I/II; 4: E domain III

After three cycles of subcloning/screening with indirect ELISA and IFA, two hybridomas secreting antibody against duck TMUV were isolated and designated as 9E4 and 4C10. The MAb 9E4 was selected for development of the bELISA as it displayed high affinity to the coating virus antigen in the preliminary indirect ELISA test. The heavy chain subclass of the 9E4 MAb was determined as IgG1 and the light chain was kappa type. Immunofluorescence and immunochemistry detection of TMUV-infected BHK-21 cells exhibited strong staining in the cytoplasm of infected cells (Fig. 1a). Cells infected with Japanese encephalitis virus or duck-origin Batai virus were stained in parallel and no positive signal was observed, suggesting that the 9E4 MAb did not react with the two arborviruses reported to infect domestic ducks in China. Using the standard PRNT in BHK-21 cell, the murine ascitic 9E4 MAb showed 90% plaque reduction (PRNT_90_) at a dilution of 1:10 and PRNT_50_ at a dilution of 1:50, demonstrating that this MAb possessed weak neutralizing activity against TMUV (see Additional file 2). Western blot analysis with the viral antigen revealed that the 9E4 MAb recognized a band with a molecular weight of 52Kd, corresponding to the E protein of TMUV (Fig. 1b). Of note, the MAb reacted with E protein only when non-reducing sample buffer without 2-mercaptoethanol was used in sample preparation for SDS-PAGE separation, indicating its epitope binding was related to the presence of disulfide bonds in the protein. Further analysis with recombinant fragments covering different domains of the E protein under non-reducing condition demonstrated that the MAb bound an epitope within domain I/II (Fig. 1c).

### Optimization of the blocking ELISA protocol

**Fig. 2:**
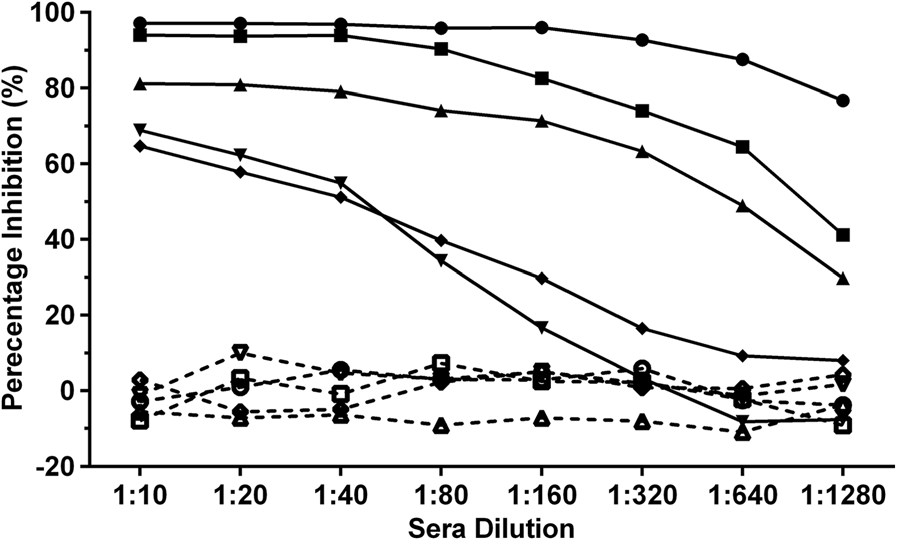
Optimization of test sera dilution. Percentage inhibition of five negative sera (dotted line) and five positive sera (solid line) was detected by the blocking ELISA at different dilutions

To determine the optimal dilution of the test serum sample in the bELISA, five positive duck sera with PRNT_50_ titers ranging from 10 to 1280 were assessed in serial dilution. As shown in Fig. 2, the PIs of antibody-negative sera did not show significant variation in serial dilution, while the PI of the positive sera decreased with dilution. Sera with lower neutralizing titer (PRNT_50_ = 10 and 20) declined rapidly for each doubling dilution, with 1:20 diluted sera producing < 60% inhibition. To ensure effective and sufficient blocking of the epitope recognized by MAb 9E4, the working dilution of the test serum samples was fixed at 1:10 in this study.

To determine the cut-off value for the bELISA, a panel of 400 duck sera lacking antibodies to TMUV was tested for non-specific inhibition of MAb binding to the coating antigen. These sera showed less than 20% plaque reduction at a dilution of 1:5 by VNT. The mean PI was − 0.65% for these sera with a standard deviation (SD) of 10.22%. The cut-off value of the test serum was set at 30% based on the criteria of “mean PI of the negative sera plus 3 × SD”, with 99% confidence of the PI< 30%. Positive serum was set as 30%. Confirmation of serum samples exhibiting a PI between 20% (X + 2SD) and 30% was required by repeating the test and it was considered to be negative if the PI was still ≤30%. Accordingly, the antibody titer was calculated as the highest dilution with a PI> 30%.

### Comparison of the blocking ELISA with virus neutralization assay

**Fig. 3:**
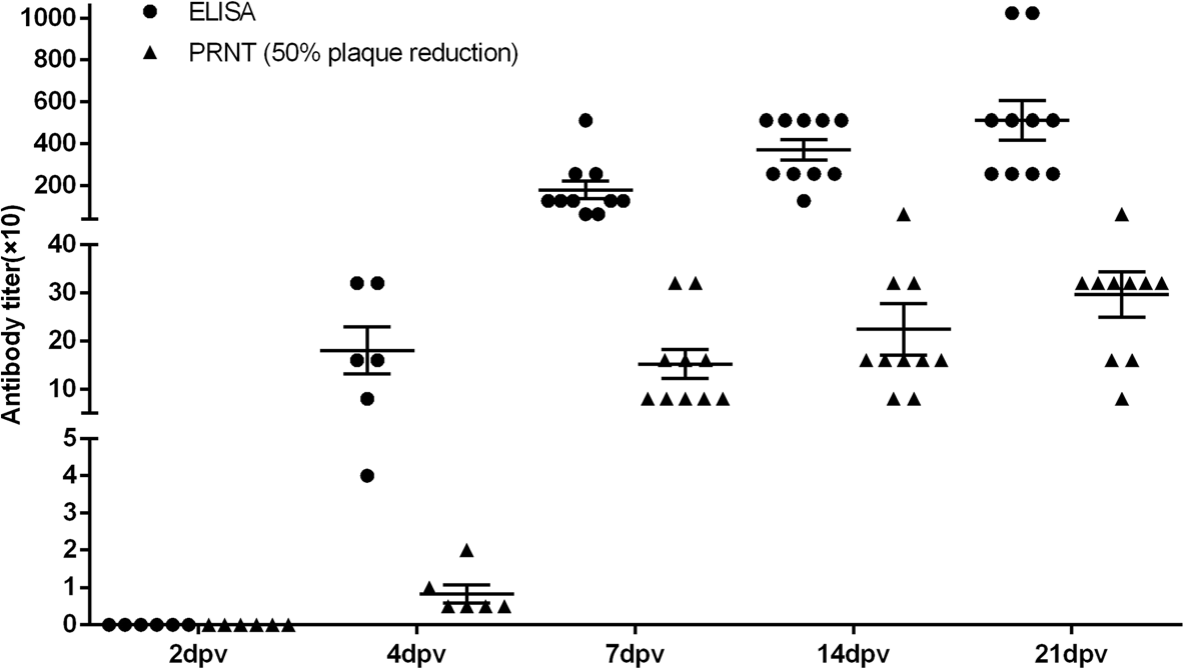
Antibody responses in ducks inoculated with attenuated duck TMUV JXSP. The serum samples were collected for antibody titer testing both by bELISA and VNT at 2, 4, 7, 14 and 21 days post vaccination. Symbols represent results from individual samples, bars indicate the mean titer values ± SE

To test the performance of the bELISA, sera collected from ducks experimentally infected with TMUV were tested. As shown in Fig. 3, the antibody response to TMUV was detectable by bELISA on day 4 after infection, with the titers ranging from 1:40 to 1:320. In parallel, these sera were found to have weak neutralizing antibodies to TMUV as detected by VNT with PRNT_50_ titers ranging from 5 to 20 (Fig. 3). Thereafter, the antibody levels determined by both bELISA and VNT increased significantly. For the sera of the five non-infected ducks, no TMUV-specific antibodies were detected by bELISA or VNT throughout this experiment. Linear regression analysis using GraphPad Prism showed a strong correlation between the serum antibody levels determined by VNT (PRNT_50_) and blocking ELISA titers, with an r^2^ value of 0.7998 (*P* < 0.001).

To exclude the possibility of non-specific inhibition of MAb binding to the coating antigen by antibodies to viruses other than TMUV, sera with antibodies to DEV, DHAV, DRV, EDSV and AIV subtypes H5 and H9 were individually tested. The mean PIs plus 3 × SD of immunized sera were lower than 20%, therefore non-specific binding to the epitope did not occur.

We next sought to test the specificity and sensitivity of the bELISA for detection in clinical samples. A total of 360 duck sera collected from different duck farms were tested by the bELISA and VNT. Among the duck serum samples, 189 sera were determined to be positive by bELISA with a PI higher than 30%, and 182 samples were considered positive by VNT with a PRNT_50_ titer ≥1:5. When the results for individual samples were compared, seven samples were detected by bELISA but not VNT, resulting in 98.05% agreement between the two techniques. The 182 VNT-positive serum samples were all detected by the bELISA. It is reasonable that the discrepant seven positive sera were detected by the bELISA as ELISA is more sensitive for antibody detection. To extend the experiment, 46 serum samples collected from a goose flock with a history of TMUV infection were tested in the same manner. Highly consistent results were obtained in that 31 sera were positive by both bELISA and VNT. Moreover, when sera collected from SPF chickens immunized with the inactivated virus were evaluated, all samples exhibited titers ranging from 1:320 to 1:2560 in the bELISA (*n* = 10). Taken together, these results indicated that the specificity and sensitivity of the bELISA were 96.37% and 100%, respectively, in comparison with the VNT. The agreement rate between these two methods was 98.32% with the kappa value 0.966.

### Application for large scale field sample detection

As shown in Table 1, all sera collected in 2009, the year before the first outbreak of duck TMUV infection, were negative while the sera collected from three laying duck flocks that recovered from the infection in 2011 showed 100% antibody positivity to TMUV, suggesting that exposure to the virus is extensive during an outbreak. This is in agreement with other studies showing that TMUV transmitted efficiently among ducks and caused severe egg drop [18, 19]. The positivity rate of the sera collected from 2012 to 2015 decreased significantly. It was reasoned to be related to the cessation of the epidemic and replacement of breeding flocks in the duck production industry. However, antibodies detected in a few samples suggest the existence of sporadic infection in duck flocks. Interestingly, duck flocks immunized with inactivated vaccine exhibited a high rate of antibody response but the positivity rate showed a significant difference.

## Discussion

Since the outbreak of duck TMUV infection in China in 2010, several serological diagnostic techniques have been reported [11–13], but they vary by antigen used and the degree of validation. In this study, a blocking ELISA was developed based on a MAb specific to TMUV, which fulfils the need for detection of kinetic antibody responses in ducks infected experimentally with TMUV or immunized with vaccine. In addition, as the assay is based on the blockade of epitope binding, it would presumably detect all types of TMUV antibodies from any host species which can bind the epitope recognized by the MAb.

The E protein is the major surface protein of flaviviruses and it plays a critical role in virus infection, as well as being a principle target of neutralizing antibodies [20, 21]. A number of anti-E protein MAbs have been successfully used in the development of ELISAs for the diagnosis of flavivirus infections [22–24]. The MAb 9E4 used in this study was mapped to recognize an epitope in the domain I/II of TMUV E protein. Results of IFA and IHC tests with virus-infected cells demonstrated that the MAb binding epitope is accessible on the virion surface (Fig. 1a). Western blot analysis revealed that the MAb 9E4 binds the epitope only when disulphide bonds in the fragment were intact without reduction by 2-mercaptoethanol (Fig. 1b and c). Since the existence of intact disulphide bonds in the E protein were shown to be necessary for induction of neutralizing antibodies in other flaviviruses [25, 26], the conformationally dependent binding of MAb 9E4 implied that the epitope might preferentially be blocked by specific neutralizing antibodies to the native E protein.

Analysis of the values of 400 negative duck serum samples with 2SD and 3SD statistical approaches yielded an optimal diagnostic cut-off value of 30% for the bELISA, which was highly consistent with the ROC analysis (see Additional file 2). In order to ascertain that the bELISA can effectively detect TMUV antibodies in birds, experimentally immunized chicken serum samples, and field-collected duck and goose serum samples were further tested. Because there is no government-approved serological diagnostic technique for TMUV infection, we chose the VNT as a reference, using the PRNT_50_ titer as criteria for positive sera. The bELISA results displayed a high level of agreement with the VNT (PRNT_50_). The discrepant seven positive duck samples detected by bELISA gave a PI in the borderline range, indicating that a low level of antibody was present in these samples. It is unsurprising that it was not detected by VNT since this technique is less sensitive than ELISA. Our blocking ELISA was able to detect TMUV-specific antibody response from 4 days post experimental infection, and the titers determined by bELISA correlated well with PRNT_50_ titers throughout the experiment (Fig. 3). Comparison of antibody levels determined by bELISA and PRNT_50_ also indicated that the former was superior in sensitivity.

Our bELISA was applied to 2359 domestic duck serum samples from semi-open duck farms in Northern China. As these samples were submitted for antibody evaluation after vaccination with inactivated avian influenza vaccines, the results are more likely to be a true reflection of the seroprevalence levels of TMUV in these farms. Antibodies to TMUV were detected in all samples in March 2011, being consistent with our earlier study [2], in which 100% antibody positivity was found in ducks after TMUV infection, supporting that TMUV is highly infectious and abundant in duck flocks during outbreaks of the disease. Several factors could contribute to the decline of positivity rates for the samples between 2012 and 2014. First, the infected duck flocks were culled or replaced as part of the duck production system. Second, owners paid more attention to biosecurity on duck farms; they did not introduce breeding eggs or ducklings from endemic areas. Importantly, high rates of antibody responses were detected in the samples collected from duck flocks experimentally immunized with autogenous inactivated TMUV vaccines. This result indicates potential use of vaccine for the control of TMUV infection in ducks, but it also presents a challenge for the differential diagnosis between ducks naturally infected by TMUV and those immunized with vaccine.

## Conclusions

The well-validated epitope-blocking ELISA is useful for a range of serological investigations of TMUV infection in multiple poultry species. It can also be used to measure the antibody responses and assess vaccine efficacy in birds after immunization.

## Additional files

Additional file 1: Recombinant plasmid construction and protein expression. (PDF 299 kb)

Additional file 2: Neutralizing activity of  MAbs 9E4 and ROC analysis of the blocking ELISA. (PDF 370 kb)

## Abbreviations

AIV: Avian influenza virus
bELISA: Blocking ELISA
BPL: Beta-propiolactone
CPE: Cytopathic effect
DAPI: 4, 6-diamidino-2-phenylindole
DEV: Duck enteritis virus
DHV: Duck hepatitis virus
DRV: Duck reovirus
EDSV: Egg drop syndrome virus
ELISA: Enzyme-linked immunosorbent assays
HRP: Horseradish peroxidase
IFA: Immunofluorescence assay
MAb: Monoclonal antibody
NDV: Newcastle disease virus
PFU: Plaque forming units
PI: Percentage inhibition
PRNT: Plaque reduction neutralization test
PRNT50: 50% plaque reduction
PVDF: Polyvinylidene fluoride
SAS: Saturated ammonium sulfate
SD: Standard deviation
TMUV: Tembusu virus
