# Supplementary material for: Development and application of a monoclonal antibody-based blocking ELISA for detection of antibodies to Tembusu virus in multiple poultry species
Source: BMC Vet Res. 2018 Jun 25;14:201. doi: 10.1186/s12917-018-1537-6 (PMC6019803; doi:10.1186/s12917-018-1537-6)
Supplement: Supplementary file 1 — Recombinant plasmid construction and protein expression. (PDF 299 kb) [file 12917_2018_1537_MOESM1_ESM.pdf]

Development of an epitope-blocking ELISA for detection of antibodies against Tembusu virus in domestic birds

BMC Veterinary Research

Lijiao Zhang, Zhanhong Li, Huan Jin, Xueying Hu,Jingliang Su

Lijiao Zhang, Institution: .Key Laboratory of Animal Epidemiology and Zoonosis, Ministry of Agriculture, College of Veterinary Medicine, China Agricultural University, Beijing 100193, China, Email: zhang62810003@126.com

Zhanhong Li, Institution: .Key Laboratory of Animal Epidemiology and Zoonosis, Ministry of Agriculture, College of Veterinary Medicine, China Agricultural University, Beijing 100193, China, Email: dy081lzh@163.com

Huan Jin, Institution:.Key Laboratory of Animal Epidemiology and Zoonosis, Ministry of Agriculture, College of Veterinary Medicine, China Agricultural University, Beijing 100193, China, Email: jinhuan0717@126.com

Xueying Hu, Institution: College of Veterinary Medicine, Huazhong Agricultural University, Wuhan 430070, China, Email: hxying@mail.hzau.edu.cn

Corresponding author: Jingliang Su, Institution:.Key Laboratory of Animal Epidemiology and Zoonosis, Ministry of Agriculture, College of Veterinary Medicine, China Agricultural University, Beijing 100193, China, Email: suzhang@cau.edu.cn; tel: +86-10-62732312.

Running title: Blocking ELISA for Tembusu virus antibody detection

Recombinant plasmid construction and Protein expression

Ectodomain, domain I/II (D I/II) and domain III (D III) gene of E protein of DTMUV JXSP were amplified by polymerase chain reaction (PCR) using the primers listed in table S1, the BamH I and Xhol I restriction sites were underlined. Three PCR products were gel-purified and digested with restriction enzymes BamH I / XhoI I followed by cloning into the expression vector pET32α (Novagen, Madison, USA) to construct the recombinant plasmid pET32/E, pET32/D I/II and pET32/D III. The plasmids were then transfected into *E. coli*transetta (DE3) (TransGen Biotech, Beijing, China). The expressed fusion protein of E, E D I/II and E D III were existed in the form of inclusion body and then preliminarily purified with washing buffer (50 mMTris-HCl, 10 mM EDTA, 0.5% Triton X-100 and 0.15 M NaCl) and denatured in lysis buffer (50 mMTris-HCl, 2 mM EDTA, 6 M Guanidine hydrochloride and 10 mMdithiothreitol) overnight at 4 ℃. The denatured proteins were then renatured in renaturation buffer (50 mMTris-Hcl, 0.2 mM EDTA, 0.5 mMArg, 1 mMGSSG, 3 mM GSH, 1 M Guanidine hydrochloride and 0.15 M NaCl) to reform the disulfide bridges and then desalinated by dialysis. The plasmid PET32α without inserted gene was performed in the same way as control. The renatured proteins were analysed by SDS-PAGE both under non-reducing and reducing condition. The SDS-PAGE anslysis showed that each renatured protein presented a clear target band and the molecular weight of each of them was slightly lower under non-reducing codition (Fig. S2), This because that the renatured protein could not completely linearized under non-reducing codition which reducing the SDS binding capacity. The reslut indicated that these recombinant proteins were successfully expressed and renatured.

Table S1 Primers used for the amplification of E ectodomain and different regions

Region	Primer name	Primer sequence	Production
E ectodomain	TMUVE F	5’-CGCGGATCCTTCAGCTGTCTGGGGATGCAG-3’	
	TMUVE R	5’-ATCTCGAGTGCTCCTTTGAGTGTTGAC-3’	1245 nt
E D I/II	TMUV E F	5’-CGCGGATCCTTCAGCTGTCTGGGGATGCAG-3’	
	TMUV D I/II R	5’-ATCTCGAGCATTCCTTTCAGCTTCAGA-3’	900 nt
E D III	TMUV D III F	5’-CGCGGATCCACCTACCCGATGTGTAGCAA-3’	
	TMUV E R	5’-ATCTCGAGTGCTCCTTTGAGTGTTGAC-3’	345 nt

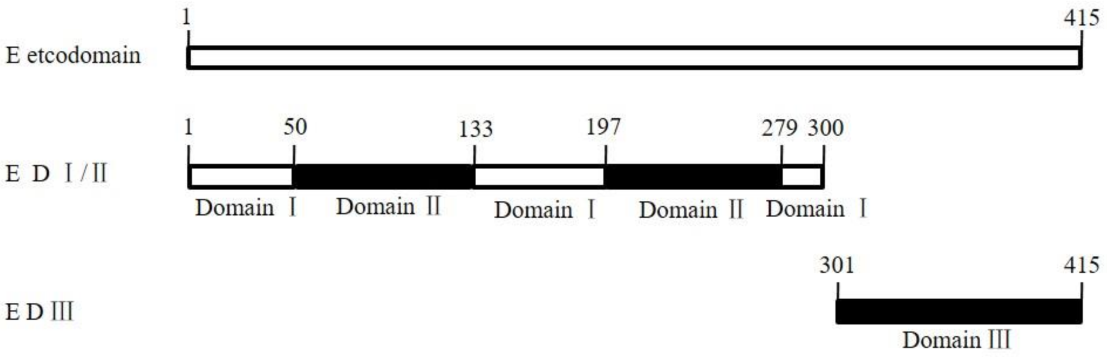

Fig. S1 Domain diagram of envelope protein of DTMUV

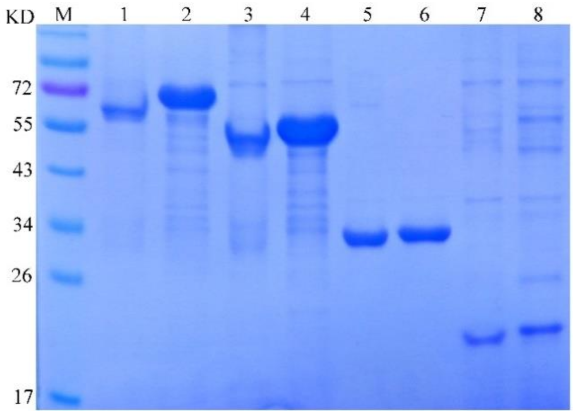

Fig. S2 SDS-PAGE analysis of renatured recombinant expression protein. (**1&2**) Pet32a-E protein; (**3&4**) Pet32a-D I-II protein; (**5&6**) Pet32a-D III protein; (**7&8**) Pet 32a tag protein; (**1&3&5&7**) Proteins under non-reducing condition; (**2&4&6&8**) Proteins under reducing condition
