# Supplementary material for: Development and application of a monoclonal antibody-based blocking ELISA for detection of antibodies to Tembusu virus in multiple poultry species
Source: BMC Vet Res. 2018 Jun 25;14:201. doi: 10.1186/s12917-018-1537-6 (PMC6019803; doi:10.1186/s12917-018-1537-6)
Supplement: Supplementary file 2 — Neutralizing activity of MAbs 9E4 and ROC analysis of the blocking ELISA. (PDF 370 kb) [file 12917_2018_1537_MOESM2_ESM.pdf]

Development of an epitope-blocking ELISA for detection of antibodies against Tembusu virus in domestic birds

BMC Veterinary Research

Lijiao Zhang, Zhanhong Li, Huan Jin, Xueying Hu,Jingliang Su

Lijiao Zhang, Institution: .Key Laboratory of Animal Epidemiology and Zoonosis, Ministry of Agriculture, College of Veterinary Medicine, China Agricultural University, Beijing 100193, China, Email: zhang62810003@126.com

Zhanhong Li, Institution: .Key Laboratory of Animal Epidemiology and Zoonosis, Ministry of Agriculture, College of Veterinary Medicine, China Agricultural University, Beijing 100193, China, Email: dy081lzh@163.com

Huan Jin, Institution:.Key Laboratory of Animal Epidemiology and Zoonosis, Ministry of Agriculture, College of Veterinary Medicine, China Agricultural University, Beijing 100193, China, Email: jinhuan0717@126.com

Xueying Hu, Institution: College of Veterinary Medicine, Huazhong Agricultural University, Wuhan 430070, China, Email: hxying@mail.hzau.edu.cn

Corresponding author: Jingliang Su, Institution:.Key Laboratory of Animal Epidemiology and Zoonosis, Ministry of Agriculture, College of Veterinary Medicine, China Agricultural University, Beijing 100193, China, Email: suzhang@cau.edu.cn; tel: +86-10-62732312.

Running title: Blocking ELISA for Tembusu virus antibody detection

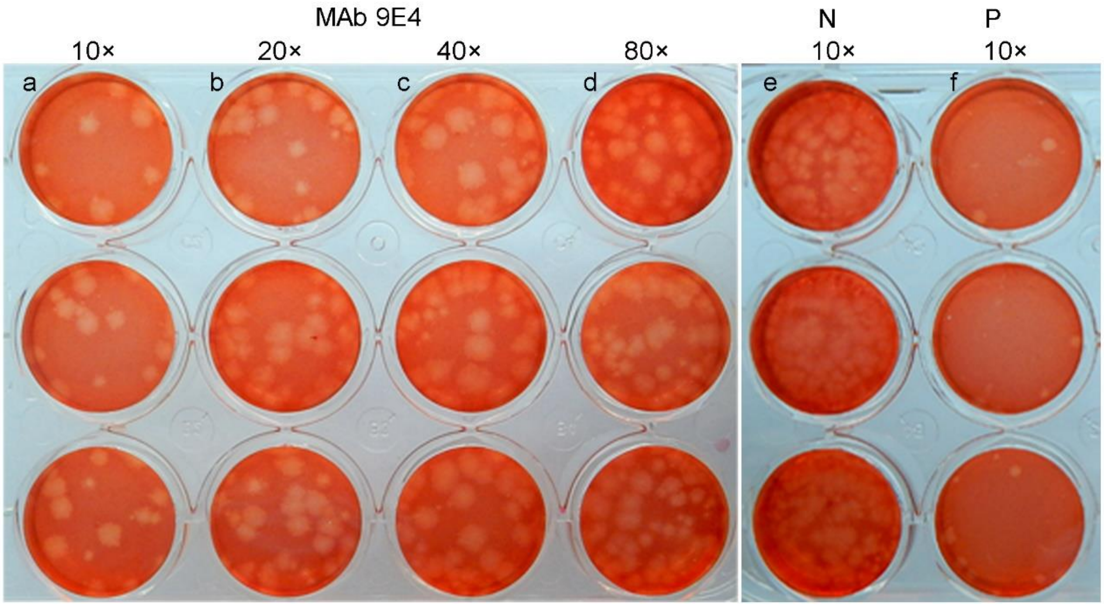

Fig. S3 Neutralizing activity of generated mAbs 9E4. (**a-d**) The mAb 9E4 was diluted 10-fold, 20-fold, 40-fold and 80-fold, respectively, to incubate with equal volume of DTMUV; (**e**) The mAb against Reovirus was diluted 10-fold to incubate with equal volume of DTMUV as negative control; (**f**) The antiserum against DTMUV was diluted 10-fold to incubate with equal volume of DTMUV as positive control

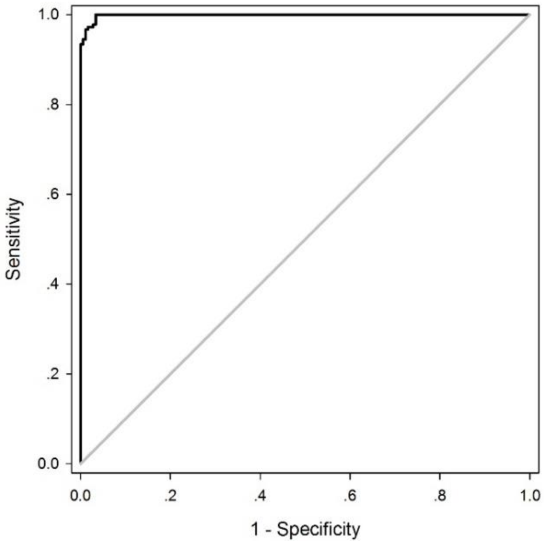

Fig. S4 ROC analysis curves for the blocking ELISA. The graph was calculated with 178 negative serum samples and 182 positive serum samples from ducks which comfirmed by PRNT. Points on the curve correspond to different cutoff values for the test. The area under the ROC curve was 0.9987

Table S2 Sensitivity and specificity of the blocking ELISA with different cut off value by ROC analysis

cut-off value	sensitivity	specificity
29.25%	100%	95.91%
29.95%	100%	96.07%
30.86%[a]	100%	96.63%
31.31%	99.45%	96.63%
31.61%	98.90%	96.63%

[a] The optimal cut-off value of the blocking ELISA was 30.86% according to the ROC analysis, yielding the maximal Yonden's index (sensitivity+specificity-1)
